# Tallow, Rendered Animal Fat, and Its Biocompatibility With Skin: A Scoping Review

**DOI:** 10.7759/cureus.60981

**Published:** 2024-05-24

**Authors:** Margaret F Russell, Manmeet Sandhu, Maddison Vail, Christa Haran, Unaiza Batool, Jonatha Leo

**Affiliations:** 1 Dermatology, Alabama College of Osteopathic Medicine, Dothan, USA; 2 Anatomical Sciences, Alabama College of Osteopathic Medicine, Dothan, USA

**Keywords:** emulsions, dermatology, natural, moisturizer, skincare, tallow

## Abstract

There is a surge in the skincare industry marketing the use of natural ingredients as efficacious agents. Although this has been popular in the Eastern hemisphere for a while, Western countries are starting to put more emphasis on naturally derived products. This paper chose to analyze the current research available on tallow, which is a solid fat derived from animals. Tallow has long been used as a neutral cooking fat, ingredient in soaps, biofuel product, and now ingredient in skincare products. The purpose of this scoping review was to look at the current research pertaining to the therapeutic benefits of tallow on the skin. Using the PRISMA Extension for Scoping Reviews (PRISMA-ScR) guidelines, a scoping review was conducted using two databases: EMBASE and PubMed as sources of evidence. The searches for studies were conducted using the following key terms: (tallow) AND (skin or dermatology or dermatitis or emulsion or cosmetics or eczema). Papers were excluded if they were not in English, if they did not mention the effects of tallow on the skin, and if they did not use tallow rendered from an animal. Date ranges and geographical locations for articles were not part of our inclusion or exclusion criteria. We focused on the following five research questions: Does the composition of tallow make it better suitable for use on skin? What is the benefit of using tallow on skin? Does tallow have therapeutic properties for skin conditions? What side effects does tallow have on the skin? Is tallow reef-safe? While there is much evidence supporting the use of tallow as an ingredient in animal feed, cooking, soaps, and biofuels, there are significant research gaps in how it can be used on human skin. Our search on PubMed and EMBASE resulted in a total of 147 studies being screened with 19 fitting our specific criteria. Of the 19 studies, there were comparative studies, basic science studies, and animal studies. After reviewing the studies to answer the objectives in this paper, we were able to find information that supported the first three objectives; however, more research is still needed. Specifically, more research is needed that is geared towards tallow as a cosmetic product in humans. The fourth objective, which was to answer the side effects of topical tallow, had the most discrepancies between the sources. The fifth objective also found supporting information; however, only two sources were found. Overall, there needs to be more research with controlled variables on the side effects of topical tallow. Different research designs that could be explored include case studies, randomized controlled trials, cross-sectional studies, and qualitative studies.

## Introduction and background

Tallow or rendered animal fat has long been used as a neutral fat for cooking, ingredient for soap products, and biofuel product, and has also been studied for its effects on the skin [1]. The composition of tallow, the possible benefits it provides for the skin, adverse effects, and preparations are of particular interest in this scoping review. Tallow is rich in triglycerides, specifically oleic acid, palmitic acid, stearic acid, and linoleic acid, thus lending to its wide range of industrial applications [2]. Tallow also contains essential vitamins, such as vitamins A, D, K, E, and B12 [2].

While there are similarities in the composition of skin and tallow, to effectively transport ingredients or substances through the skin and how tallow could be beneficial to the skin, one must have a basic understanding of the physiology of the skin. The outermost layer of skin, the stratum corneum, consists of non-nucleated keratinocytes arranged in a bricklike fashion [3]. While this is crucial for protecting humans from possible pathogens, penetrating the stratum corneum poses the largest hurdle for absorbance of topical products into the skin. The keratinocytes are connected through adhesions between various lipid components [3]. Specifically, the inner layer of the stratum corneum is a lipid-rich matrix. The main lipids found in the skin's protective barrier, stratum corneum, are cholesterol, free fatty acids, and ceramides [4]. These properties allow for the keratinocytes to be strengthened by a rich lipid matrix, creating a semipermeable barrier.

Cholesterol, free fatty acids, and ceramides are synthesized in the body to provide the skin with the vital building blocks it needs. All are essential for the skin; however, the body has a finite amount of lipid production, and thus there is a need to provide the body with essential fatty acids [5]. This can be especially true in skin disorders where lipid barrier function may be compromised [5]. Topical application of physiological lipids can improve permeability barrier homeostasis and has been used to treat cutaneous diseases [4]. The delivery of essential lipids can come from a variety of different places, one being through moisturizing products readily absorbed through the skin [6]. While the delivery and need for different nutrients varies from individual to individual, overall, the right formulation depends on what is missing in the skin. For example, in patients suffering from atopic dermatitis, ceramides must be replenished [7]. Furthermore, the tight junctions between the cells of the stratum corneum comprise the main barrier to penetration or entry of substances. Research has reported that small molecules, specifically below 500 Dalton, are able to pass transdermally [8]. There are multiple routes of absorption that can be taken advantage of, but the main route of absorption, through the stratum corneum, is paracellularly [9]. Parameters such as these are used when trying to formulate products for the skin, whether it is for drug delivery or cosmetic purposes. Specifically, fatty acids and triglycerides are often used to develop vehicle delivery systems such as lipid nanocarriers [10]. It is reported that fatty acid uptake into keratinocytes is transport-mediated and temperature-sensitive. Specifically, it is reported that there is a higher specificity for the uptake into keratinocytes with fatty acids, linoleic acid, and arachidonic acid [4]. With fats being an integral part of keeping the protective layer of our skin together, pushing more research into high fat containing moisturizers, such as tallow, could prove to be useful. With a product like tallow, which has shown its benefits through its innately occurring composition, it is important to learn more about the use of this natural, biocompatible product because of the innate benefits it could provide.

Additionally, we must consider the attractiveness of a product like tallow as it is naturally derived from animals and how this plays a key role in the rise of its use in the skincare industry. With the rise in demand for “natural” products in skincare, an increasing number of products are being marketed as “natural” or “clean,” calling into question what these terms really mean [11]. These terms can be used as a marketing ploy by the cosmetic industry to deem synthetic or lab-created ingredients as “toxic” or “chemicals” that are harmful to the skin. The words “clean” and “natural” are unregulated marketing terms that can be used to describe any product free of ingredients deemed unacceptable by a particular company. The most common ingredients targeted include parabens, petrolatum, formaldehyde releasers, and phthalates because of possible hormone disruption [11]. Companies such as Toups and Co. found a way to use tallow as the main ingredient in their moisturizers. Products like these are part of the clean beauty movement and free from artificial colors, synthetic fragrances, chemicals, and petroleum [12]. The product is also marketed as being non-comedogenic, which makes an ingredient like tallow beneficial to many skin types. The claim that tallow has better biocompatibility with human skin compared to plant-based and manufactured products leads us to ask the following questions: How does tallow benefit skin? What research supports the argument that tallow provides beneficial properties to the skin? The uses of tallow pertaining to skin have been explored in a limited number of studies, making it challenging to assess the potential uses of tallow in products such as skincare, cosmetics, and medication. Furthermore, no literature review articles could be found that summarized the studies conducted with tallow on human skin. Therefore, the aim of this scoping review was to report the evidence-based research that pertains to tallow and its use on human skin. We aimed to answer the following research questions: Does the composition of tallow make it better suitable for use on skin? What is the benefit of using tallow on skin? Does tallow have therapeutic properties for skin conditions? What side effects does tallow have on the skin? Is tallow safe for aquatic life?

## Review

Materials and methods

The published methods outlined by the PRISMA Extension for Scoping Reviews (PRISMA-ScR) guidelines were used in this scoping review [13]. Two databases were searched: PubMed and EMBASE. Only articles in English, those that discussed the topical dermatological effects of tallow, and studies that used animal-rendered tallow were used. Exclusionary criteria included papers that were not in English and papers that did not mention tallow for topical use or used tallow derived from a non-animal source. To carry out the search, the following key terms were used: (tallow) AND (skin or dermatology or dermatitis or emulsion or cosmetics or eczema). Microsoft Excel (Microsoft, Redmond, WA) was used to organize the searches. The titles and abstracts were used by the reviewers to screen the papers. If they did not mention tallow being used on the skin in some capacity, they were omitted. The papers were subsequently discussed and summarized among the reviewing team members. All search-generated papers were then sorted and selected for exclusion or inclusion into the scoping review based on the previously mentioned criteria. Duplicates were also accounted for. All the results were then summarized with respect to answering the scoping reviews’ research questions.

**Figure 1:**
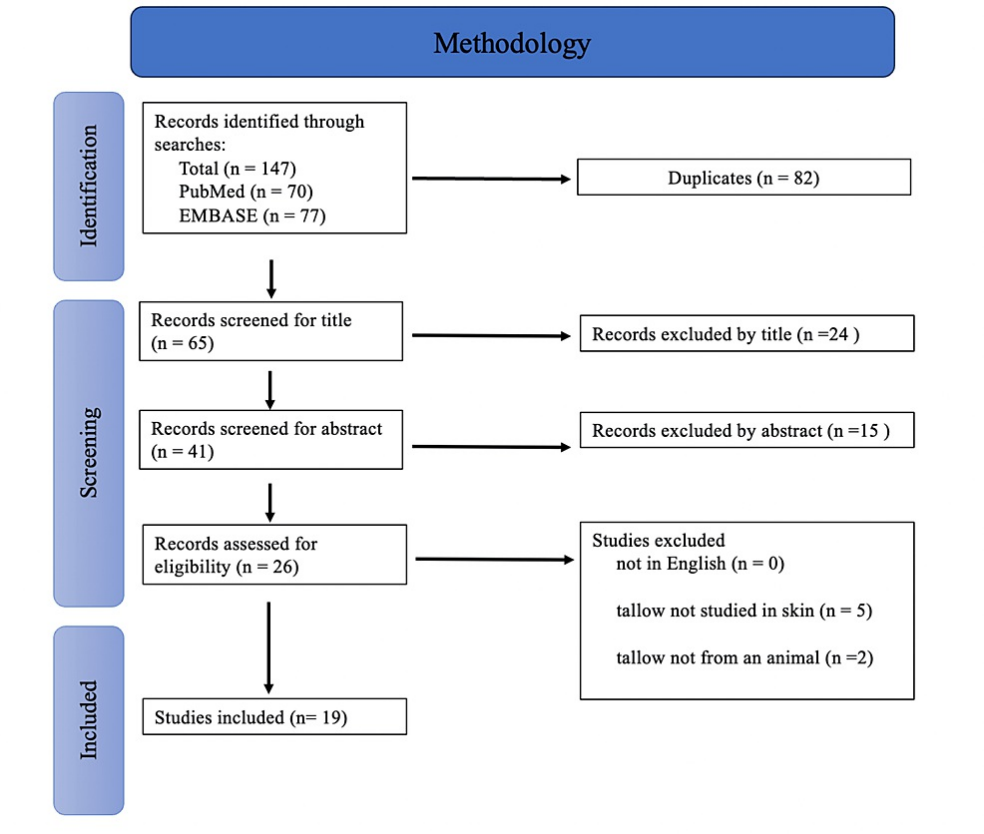
Flow chart detailing results of the search per PRISMA guidelines. PRISMA, Preferred Reporting Items for Systematic Reviews and Meta-Analyses

Figure 1 outlines the specific search methods. Through the searches, 147 articles were selected for further investigation: 70 from PubMed and 77 from EMBASE. Duplicates were accounted for, leaving 65 records total to screening; 24 papers were not used to title, 15 were not used due to abstract, and 26 were screened for eligibility. Finally, 19 papers were chosen for this scoping review.

Results

Table 1 highlights the 19 records included in the scoping review. Specifically, Table 1 shows the aims of the research, summary of the findings, objectives answered, and the type of study conducted.

**Table 1:** Summary of articles included in the review. ALA, alpha-linolenic acid; CNS, central nervous system; GABA, gamma-aminobutyric acid; GARD, genomic allergen rapid detection; IFA, incomplete Freund's adjuvant; IR, insulin resistance; LC50, lethal concentrations; NEFA, non-esterified fatty acids; POE-T, polyethoxylated tallow amine; SLM, solid lipid microsphere; TAG, triacyclglycerols

Article Name	First Author	Aim of Research	Findings	Objective Addressed	Design of Study
The acute oral toxicity and primary ocular and dermal irritation of selected N-alkyl-2-pyrrolidones	Ansell and Fowler, 1988 [14]	To gather the dermal and ocular irritation associated with topical application of tallow on rabbits.	N-alkyl-2-pyrrolidones toxicity is dependent on the alkyl substitution size. N-cyclohexyl showcased the greatest level of toxicity to all fields tested: acute oral, CNS, dermal, and eye. Acute oral toxicity was found to be dependent on hydrophobicity. CNS toxicity was determined by the parent compound 2-pyrrolidone, which is a precursor to GABA. The irritation potential of N-alkyl-2-pyrrolidones on the skin and eyes follows a similar trend as acute oral toxicity. Lower alkyl substituents generally have low dermal irritation potential and reversible ocular effects.	What side effects does tallow have on the skin?	Animal study
Safety assessment of ammonium hectorites as used in cosmetics	Becker et al., 2013 [15]	To gather the dermal and ocular irritation associated with topical application of tallow and other ingredients on animals and humans.	Disteardimonium hectorite, dihydrogenated tallow benzylmonium hectorite, stearalkonium hectorite, and quaternium-18 hectorite are safe to use in current cosmetic practice. Dihydrogenated tallow benzylmonium hectorite is assumed to be safe if used in a similar form and concentrations as the aforementioned ammonium hectorites, resulting in a similar determination of safety.	What side effects does tallow have on the skin?	Animal study
A glyphosate micro-emulsion formulation displays teratogenicity in Xenopus laevis	Bonfanti et al., 2018 [16]	To gather the effect of tallow and other compounds on Xenopus levis.	Lyphosate-based herbicides residues in aquatic environment have no embryolethal effect but do show teratogenic, craniofacial, and eye malformation effects on Xenopus levis embryo.	Is tallow safe for aquatic life?	Animal study
Effects of abomasal lipid infusion on liver triglyceride accumulation and adipose lipolysis during fatty liver induction in dairy cows	Brickner et al., 2009 [17]	To study the efficacy of tallow and linseed oil transfusions in increasing the plasma fatty acid concentration.	Abomasal infusion of linseed oil primarily increased ALA levels in the serum, without significant effects on adipose tissue lipolysis or plasma NEFA concentration. Linseed oil alone resulted in more basal and stimulated fatty acids than tallow or the combination of the two.	Does the composition of tallow make it better suitable for use on skin?	Basic science
Toxicity of 19 adjuvants to juvenile Lepomis macrochirus (bluegill sunfish)	Haller and Stocker, 2003 [18]	To analyze the toxicity of tallow amine in aquatic herbicides.	The toxicity of 19 adjuvants to bluegill sunfish was tested, and the two ethoxylated tallow amine products showed the most toxic with LC50 levels of 1.6 and 2.9 ppm. The ethoxylated tallow amines were found in shallow waters less than 10 cm only. The alcohol/glycol-based surfactants fell between LC50 values of 4.0 and 11.6 ppm. Polysiloxane-based surfactants had LC50 values between 18.1 and 29.7 ppm. Limonene-based surfactants had LC50 levels of 10.2 and 30.2 ppm. The methylated seed oil with emulsifier had an LC50 value of 53.1 ppm. The two acid/buffer utility adjuvants had LC50 values of 60.8 and 221 ppm. Surfactants that were found in shallow water (<10cm) were found to be the most toxic, and fish that did not move to deeper water (>10cm) could not avoid the lethal concentrations.	Is tallow safe for aquatic life?	Comparative study
Herbal soap formulation using plant extracts and essential oils	Khan et al., 2018 [19]	To analyze the wound healing effects of herbal soaps that comprise tallow.	The herbal soaps with tallow as a main ingredient showed higher antibacterial efficacy in comparison to the alkaline soaps. The herbal soap that contained essential oil and soluble compounds showed the most efficacy.	What is the benefit of using tallow on skin?	Basic science
Assessment of a stable cosmetic preparation based on enzymatic interesterified fat, proposed in the prevention of atopic dermatitis	Kowalska et al., 2017 [20]	To study the efficacy of tallow in helping with atopic dermatitis and psoriasis.	The tested emulsions with mutton tallow and walnut oil blends were shown to be effective formulations for improving skin hydration.	What is the benefit of using tallow on skin?	Comparative study
Enzymatically interesterified fats based on mutton tallow and walnut oil suitable for cosmetic emulsions	Kowalska et al., 2015 [21]	To analyze mutton tallow emulsion's efficacy on sensory and moisturizing properties on respondents.	Interesterification of mutton tallow and walnut oil modified fats show potential for application in the cosmetic industry, particularly as a base for emulsions due to their contribution to the stability of emulsion systems and enhanced moisturization properties for the skin. The emulsion blend with 13% water received the highest sensory profile ratings from the respondents.	Does the composition of tallow make it better suited for use on skin?	Comparative study
Physicochemical characterization and evaluation of emulsions containing chemically modified fats and different hydrocolloids	Kowalska et al., 2020 [22]	To compare tallow to pumpkin seed oil on their positives and negatives when applied topically.	Interesterified tallow and pumpkin seed oil of the blends when incorporated into food emulsions offer beneficial nutritional properties. In cosmetic emulsions, they can contribute to proper hydration and improved skin condition. The inclusion of pumpkin seed oil, which is rich in unsaturated fatty acids, adds attractiveness.	Does the composition of tallow make it better suitable for use on skin?	Comparative study
Quality of emulsions based on modified watermelon seed oil, stabilized with orange fibres	Kowalska et al., 2022 [23]	To gather information on properties of different emulsions with watermelon seed oil and mutton tallow.	Emulsions with higher concentrations of mutton tallow were found to be the most emulsion-stable and color-stable and had the highest moisturizing effect. Emulsions containing both xanthan gum and orange fiber as thickeners showed more favorable parameters compared to emulsions with only one viscosity modifier. Further studies need to be completed to determine sensory evaluation of mutton tallow.	What is the benefit of using tallow on skin?	Basic science
NCM 1921, a mixture of several ingredients, including fatty acids and choline, attenuates atopic dermatitis in 1-chloro-2,4-dinitrobenzene-treated NC/Nga mice	Lee et al., 2020 [24]	To study the efficacy of tallow in helping with atopic dermatitis and psoriasis.	NCM 1921, a mixture contain beef tallow among other fats, has potential as a therapeutic agent for atopic dermatitis, potentially serving as a replacement for corticosteroids or as a adjunct treatment. NCM 1921 improves symptoms, suppresses dermal lesions, reduces inflammatory cells, lowers IgE levels, regulates T cell response, and enhances skin barrier function.	Does tallow have therapeutic benefits to skin?	Animal study
An integrated transcriptomic- and proteomic-based approach to evaluate the human skin sensitization potential of glyphosate and its commercial agrochemical formulations	Lindberg et al., 2020 [25]	To analyze the possible irritation and photosensitivity effects of tallow.	The toxicological assessments conducted by the European Food and Safety has identified scientific evidence supporting a higher toxicity of POEA (polyethylated tallow amine) in several toxicological endpoints, including skin irritation and sensitization, when compared to the glyphosate alone (banned by the European Union). GARD skin assay classified tallow as a skin photosensitizer.	What side effects does tallow have on skin?	Basic science
Toxicology and human health risk assessment of polyethoxylated tallow amine surfactant used in glyphosate formulations	Martens et al., 2019 [26]	To analyze the possible irritation effects of tallow and how the effects change upon dilution with water.	Concentrated POE-T was very irritating and sensitizing to the skin, eyes, and respiratory and gastrointestinal tracts. The irritation and sensitization potential of POE-T diminish significantly upon dilution with water. POE-T was deemed safe and non-irritating in accordance with its toxicity profile, occupational risk assessment, and food risk assessment.	What are the side effects of tallow on the skin?	Basic science
Effects of intravenous triacylglycerol emulsions on hepatic metabolism and blood metabolites in fasted dairy cows	Mashek et al., 2005 [27]	To analyze the efficacy of tallow increasing plasma fatty acid concentrations and the role it plays in suppressing lipolysis.	Plasma TAG concentrations reduced most with linseed oil treatment. NEFA and glycerol concentrations showed no significant difference between fatty acid infusions though. Plasma glucose concentrations were greatest with tallow treatment. Total oxidation increased most with fish oil treatment. Hepatic TAG increased most with fish oil treatment.	Does the composition of tallow make it better suitable for use on skin?	Basic science
Surface modified solid lipid microparticles based on homolipids and Softisan® 142: Preliminary characterization	Nnamani et al., 2010 [28]	To gather information on the compositional properties of tallow.	SLMs have the most optimal syringeability and stability at temperature between 4 and 6 degrees Celsius and injected with a 27 G needle. Tallow fat shows the lowest crystallinity, while Softisan 142 showed the highest crystallinity. Addition of polysorbate 80 to SLMs reduces enthalpy, mostly to the goat fat.	Does the composition of tallow make it better suitable for use on skin?	Basic science
Induction of hyperlipidemia by intravenous infusion of tallow emulsion causes insulin resistance in Holstein cows	Pires et al., 2007 [29]	To analyze the efficacy of tallow increasing blood fatty acid concentrations compared to a control.	Intravenous glucose tolerance tests showed IR in Holstein cows due to increased lipid levels. This IR affects cows during the time of giving birth through elevation of NEFA, which causes adipocytes to become more resistant to insulin. This, in turn, increases the concentration of NEFA in the blood, potentially leading to metabolic disorders related to energy imbalance. Tallow was used in emulsion to deliver the NEFA. This study suggests how specific diets can either support or hinder the cows’ ability to complete lactation.	Does the composition of tallow make it better suitable for use on skin?	Animal study
High yield lipase-catalyzed synthesis of Engkabang fat esters for the cosmetic industry	Abd Rahman et al., 2011 [30]	To compare fat to fat esters in increasing skin hydration and tallow's overall effect on boosting hydration.	Acute moisturizing tests showed that Engkabang fat ester significantly increased skin hydration in comparison to the Engkabang fat. The properties and characteristics of the esters produced were found to be suitable for use as cosmetic ingredients.	What is the benefit of using tallow on skin?	Basic science
Different adjuvanticity of incomplete Freund's adjuvant derived from beef or vegetable components in melanoma patients immunized with a peptide vaccine	Rosenberg et al., 2010 [31]	To discuss the discontinuation of tallow-derived oleic acid in vaccine for melanoma patients and its outcomes.	There was a decrease from 60% to 10% in generation of peptide-specific T cells with vegetable-derived IFA in comparison to beef-derived IFA. Decreased immunogenicity of vegetable-derived IFA was noted with a decrease in skin reactions. Beef-derived LFA contains more adjuvants noted by the improved IL-2 response of patients with metastatic melanoma who received the vaccination.	What side effects does tallow have on the skin?	Basic science
Enzymatically modified fats applied in emulsions stabilized by polysaccharides	Woźniak et al., 2020 [32]	To compare tallow to hemp seed oil in their composition and the effects this plays on topical application.	Using interesterified fats as a fatty base in cosmetic emulsions results in stable formulations that can be adjusted to the specific means of use, such as a skincare product. Emulsions based on interesterified fats were compared to emulsions with non-interesterified fat.	Does the composition of tallow make it better suitable for use on skin?	Basic science

The research questions used in this project were answered by the found articles, and the results of that are detailed in the following sections.

Does the Composition of Tallow Make It Better Suitable for Use on Skin?

In one study, researchers aimed to formulate a product that had the highest ratio of monoacylglycerols (MAGs) and diacylglycerols (DAGs). The emulsion system was formulated with mutton tallow and walnut oil. These systems were then used and reviewed by 83 respondents. The participants judged the emulsions based on their moisturizing properties. Overall, the study concluded that creating products with high ratios of MAGs and DAGs could prove to be useful in skincare [21]. Another research project also reported tallow and pumpkin seed oil as moisturizers with xanthan gum or carboxymethylcellulose as thickeners [22]. These moisturizers were studied since they have high thermal and oxidative stability and are stable at room temperature [22]. This study showed that the highest amount of triacylglycerol fraction was in the mixtures with the highest pumpkin seed oil and lowest amount of tallow [22].

Another study looking at the composition of various tallow emulsions showed that an emulsion with tallow and linseed oil transfusion increased plasma non-esterified fatty acids (NEFA). However, linseed oil alone resulted in more basal and stimulated fatty acids than tallow alone or in combination. When looking at the fatty acid composition of tallow, it contains a proportionally small amount of polyunsaturated fatty acids, linoleic acid (C18:2n-6) and alpha-linolenic acid (C18:3n-3) [17]. Furthermore, a study performed an infusion of tallow into an experimental group of cows with elevated blood NEFA. The results showed that with the infusion of tallow, the fatty acid plasma concentration increased from 79 ± 7 to 295 ± 9, indicating a 3.7-fold increase of fatty acids that were able to enter the bloodstream [29]. A different study interested in the usefulness of tallow as a vehicle reported that using tallow to increase the saturated fatty acids in an infusion led to decreased lipolysis in the adipose tissue of fasted dairy cows [27]. The research conducted by Nnamani et al. sought to report the use of tallow, specifically its lipid matrix, as a possible vehicle for poorly water-soluble drugs. Using solid lipid microparticles (SLMs), a drug carrier composed of lipids, they reported that tallow had a melting point of 54.5°C, with an enthalpy of -5.067 mW/mg, and that its lipid matrix could be a potential SLM [28]. The idea of tallow as a possible vehicle for oral drug delivery can be used to explore other delivery routes such as topically on skin.

What Is the Benefit of Using Tallow on the Skin?

Wozniak et al. report that tallow, a waste product from the meat industry, can be enzymatically modified with hemp seed oil to increase its polyunsaturated fat content. They also reported that the addition of modifiers, such as xanthan gum and scleroglucan, for stabilization of tallow emulsions showed no clear differences in terms of stability; thus, the addition was not justified [32] Their work provided a model system that could be modified for use in the industry of choice such as skincare because of the fat content. Additions of xanthan gum and orange fibers improved the moisturizing properties of tallow emulsions. The addition of orange fibers was to stabilize the emulsion from phase separation. Xanthan gum was also used to stabilize the emulsion and determine the ratio of ingredients that provided the most moisturizing benefit. However, the study concluded that tallow alone had the most moisturizing impact when applied to the skin [23]. Additionally, the moisturizing properties of Engkabang fat esters, which are derived primarily from tallow, showed increased skin hydration. There was an increase of 4.7%, 23.2%, 38.4%, 44.4%, and 47.2% of skin hydration following 30, 60, 90, 120, and 180 minutes of application, respectively. However, when Engkabang fat was applied, non-esterified, there was a decrease of 47.1% in hydration at 30 minutes, but after the surface layer was absorbed, there was an increase of 8.7% in hydration after 180 minutes [30]. Overall, the tallow fat esters showed significantly more increase in hydration than tallow fat [30]. Tallow for wound healing was studied through the use of a herbal soap, with tallow as an ingredient. The study specifically looked at this tallow-based soap as an anti-infective agent for wound healing versus non-tallow-based soap. The tallow-based soap was found to be efficacious as a disinfectant. It also did not cause the typical amount of dryness experienced with soaps and surfactants [19]. Overall, the aforementioned research discovered tallow to play a part in increasing hydration and moisture in the skin, along with having anti-microbial properties.

Does Tallow Have Therapeutic Properties for Skin Conditions?

The focus of a comparative study was to study the effects of a topical emulsion that had the main ingredient as tallow. Specifically, they concluded that the use of high fat containing emulsions could prove to be helpful in atopic dermatitis or psoriasis. The study had 78 participants, who reported that the moisturizing properties of the emulsion with tallow helped their skin [20]. Lee et al. also showed the usefulness of a high fat containing tallow emulsion in providing symptomatic relief to mice with atopic dermatitis. This study also showed the impact of the tallow mixture, which decreased IgE levels in mice that initially had high IgE levels due to pruritus. There was also a decrease in mast cells and B-cell markers [24].

What Side Effects Does Tallow Have on the Skin?

With a commonly used solvent, 2-pyrrolidone, Ansell and Fowler classified tallow as being a severe irritant to the skin as it caused erythema and edema. However, it was a moderate irritant to the eye and resulted in corneal effects, and though it did cause iritis and conjunctivitis when applied directly to the eye, it was completely cleared by day 7 [14]. A study looking at the direct effects of tallow on animal skin reported that tallow, in a saline solution, topically applied to intact or abraded skin of rabbits for 24 hours did not cause irritation. Also, when it was applied to their eyes following a rinse or no rinse, there was no irritation. Also, when applied topically to the skin of guinea pigs, it did not cause any type of hypersensitivity reaction in guinea pigs, and there was no associated genotoxicity or carcinogenicity [15]. Lindberg et al. [25] and Martens et al. [26] explored the effects of the tallow amines on skin. Polyethoxylated tallow amine (POE-T) was not reported to cause significant irritation to a specific organ or body system after repeated exposure through oral and respiratory consumption. However, a GARD skin assay also classified tallow as being a skin sensitizer, which increases risk of photosensitivity-related irritation [25]. Marten et al. concluded that concentrated POE-T was very irritating to skin, corrosive to the eyes, and sensitizing to the skin. Roughly, a 70% concentrated tallow causes oral, skin, eye, and reproduction toxicity in rats, rabbits, guinea pigs, and dogs. The irritation and sensitization potential of POE-T diminish significantly upon dilution with water. This study also showed that there was severe irritation with 78% of POE-T on the skin of rabbits within 24 hours of contact. However, erythema associated with this resolved within 72 hours. Tallow has been categorized as dangerous and as a compound that can cause serious eye damage as studied in rabbits. However, there is no reproduction toxicity or embryo-fetal development toxicity associated with topical tallow according to this study [26].

One study discussed that vaccine was given to patients with melanoma, which had oleic acid extracted from tallow derived from animal fat. However, the oleic acid was substituted to be extracted from olives due to the fear that there may be a possibility of transmitting prions into the blood from tallow derived from animal fat and may transmit bovine spongiform encephalopathy [31]. However, after switching the immunization, clinical trials showed that the initial vaccine was more effective in melanoma patients. The researchers found that there were less severe reactions with the formulation using the oleic acid extracted from tallow than olives. They described the possible decrease in reactivity could be due to contamination in the beef-derived formulations, but it was not entirely clear [31].

Is Tallow Safe for Aquatic Life?

A study conducted by Haller and Stocker investigated the effects of tallow-derived products on fish. Specifically, ethoxylated tallow amines in herbicides on the scales or skin of bluegill sunfish were shown to be the most harmful for the fish [18]. Another study showed that tallow in aquatic environment has teratogenic effects on microbiota such as Xenopus levis [16].

Discussion

To understand the research gaps in the use of tallow, rendered animal fat, and its use on skin, this scoping review sought to answer these five questions; Does the composition of tallow make it better suitable for use on skin? What is the benefit of using tallow on skin? Does tallow have therapeutic properties for skin conditions? What side effects does tallow have on the skin? Is tallow safe for aquatic life? These research questions were formulated based on the reported uses of tallow and then tailored to fit our specific interests: tallow and its effects on skin. The final question, is it safe for aquatic life, was included to create a more comprehensive understanding on the uses of tallow in skincare products and how those could have an effect on environments humans commonly come in close contact with. The searches of (tallow) AND (skin or dermatology or dermatitis or emulsion or cosmetics or eczema) revealed that there are significant research gaps in the uses of tallow on human skin. Of the total 147 research articles that were found, 19 fit our inclusion and exclusion criteria. The results of our searches in PubMed and EMBASE showed that most published evidence of using tallow on the skin are lacking human subjects. To better understand the impact of tallow on skin and its utility in skincare, more studies conducted directly on human skin are needed. Additionally, there were comparative studies and animal studies that reported results on the uses of tallow. These studies can help close the research gaps on the specifics of tallow and its use on skin. They also allow us to learn more about the possible benefits and consequences of tallow on the skin. To help continue to answer the proposed research questions of this scoping review and expand the overall knowledge of the use of tallow on human skin, different research study designs need to be explored. Specifically, case studies, randomized controlled trials, cross-sectional studies, and qualitative studies can be used.

First, we asked the question: does the composition of tallow make it better suitable for use on skin? Looking at the composition of tallow and the protective barrier of the skin that must be penetrated, tallow looks to be biocompatible. Tallow is rich in triglycerides, specifically oleic acid, palmitic acid, stearic acid, and linoleic acid, thus lending to its wide range of industrial applications. Tallow also contains essential vitamins, such as vitamins A, D, K, E, and B12 [2]. While there is a lack of direct research of the exact mechanism that tallow uses to cross the barrier and penetrate the deeper layers of the skin and how it works on the skin, the literature and research on the two separately provided strong evidence of tallow’s biocompatible nature. The main lipids found in the skin's protective barrier, stratum corneum, are cholesterol, free fatty acids, and ceramides [4]. Specifically, research conducted by Kim et al. reported that palmitic acid C16:0, steric C18:0, palmitoleic acid (C16:1), C18:1, linoleic acid (C18:2), and (all-cis)-11,14,17-eicosatrienoic acid (ETA, C20:3n-3) were the main fatty acids components of the epidermis [33]. This unique lipid composition of the epidermis helps structurally support the keratinocytes of the stratum corneum [34]. While useful for providing external protection, penetrating it poses the largest hurdle to cross when trying to deliver products to the skin. Research reports that the main route of absorption through the stratum corneum is paracellularly [34]. This mechanism is used for delivery of various topical drugs in the pharmaceutical and cosmetic industries. To do this, chemical enhancers are added along with the active ingredients to enhance penetration and absorption into the skin [35]. Specifically, this commonly comes in the form of emulsions, which are mixture of two substances that are usually immiscible transformed to be homogenous [36]. Within the scope of our specific research question, tallow can be used as the oil portion of emulsions. We saw the use of tallow in emulsions through the form of ointments and delivery of vaccines.

The research conducted by Nnamani et al. reported the use of tallow, specifically its lipid matrix, as a possible vehicle for poorly water-soluble drugs. They reported that bovine tallow lipid matrix is highly crystallized or solid in nature, making it difficult to load with drugs of interest. However, the addition of less crystallized lipid matrices such as, Phospholipon® 90G, to the bovine tallow matrix showed to be more useful for drug carrying in topical solutions. Also, they reported that tallow had a melting point of 54.5°C with an enthalpy of -5.067 mW/mg and that its lipid matrix could be a potential SLM, a drug carrier composed of lipids with the addition of Phospholipon® 90G [28]. Another study used walnut oil and mutton tallow to form different emulsion systems and tested them on 83 participants. Naturally occurring tallow has a high concentration of fatty acids, predominantly oleic acid, palmitic acid, and stearic acid in descending order [9]. The researchers aimed to formulate a product that had the highest ratio of MAGs and DAGs. The participants judged the emulsions based on their moisturizing properties. Overall, the study concluded that creating products with high ratios of MAGs and DAGs could prove to be useful in skincare [21]. Such research allows us to see that tallow could be a potential key ingredient in the cosmetic industry.

Emulsions are also used in the delivery of vaccines, specifically microemulsions. These types of emulsions are an important area of research because it is critical to choose a vehicle that does not react with human cells as the consequences could outweigh the benefits of a vaccine [36]. Pires et al. performed an infusion of tallow into an experimental group of cows with elevated blood NEFA. The results showed that with the infusion of tallow, the fatty acid plasma concentration was 295 ± 9. Compared to the fatty acid plasma concentration of 79 ± 7, there was over a 3.7-fold increase of fatty acids that were able to enter the bloodstream. This illustrates the ability of tallow to get into the bloodstream with minimal reactivity. Additionally, tallow may increase the concentration of fatty acids that are able to penetrate the skin and enter the bloodstream, which may facilitate the production of fatty acids in the epidermis [29]. Another study on the usefulness of tallow as a vehicle reported that using tallow to increase the saturated fatty acids in an infusion led to decreased lipolysis in the adipose tissue of fasted dairy cows [27]. An increase in saturated fatty acids, such as in this treatment, may have caused increased plasma insulin, glucose, and NEFA, and suppression of lipolysis in adipose tissue, suggesting that applying tallow topically or as a vehicle may have the potential to decrease the breaking down of fatty acids in the epidermis [27]. This poses another possible research gap as more evidence would be needed to support the reactivity of tallow with human cells when used as a vehicle in vaccines. Another study looking at the composition of various tallow emulsions showed that an emulsion with tallow and linseed oil transfusion increased plasma NEFA. However, linseed oil alone resulted in more basal and stimulated fatty acids than tallow alone or in combination. When looking at the fatty acid composition of tallow, it contains a proportionally small amount of polyunsaturated fatty acids, linoleic acid (C18:2n-6) and alpha-linolenic acid (C18:3n-3), which means it may be less effective in increasing the fatty acid content in the epidermis [17]. This suggests that additions to tallow may make it more beneficial for the skin rather than using tallow alone.

Research also reported the use of tallow and pumpkin seed oil as moisturizers, with xanthan gum or carboxymethylcellulose as thickeners. These moisturizers were studied since they have high thermal and oxidative stability and are stable at room temperature. This study showed that the highest amount of triacylglycerol fraction was in the mixtures with the highest pumpkin seed oil and lowest amount of tallow. There was also a significant droplet size increase in emulsion with mostly tallow, which indicates it is a thick substance. Tallow was also observed to be a lot stickier than pumpkin seed oil, which may deter patients from applying tallow topically [22]. These results again suggest that there are substantial research gaps, specifically in the most beneficial composition and use of tallow in various emulsions. More research would help us understand this relationship and mechanism, specifically the mechanism that tallow uses to cross the barrier and penetrate the deeper layers of the skin. One study that may help understand this relationship more is a randomized controlled trial. This type of study helps answer the following question: how effective treatments are? [37]. With this type of design, different emulsions of tallow could be used and tested to understand the penetration and hydration it may possess compared to other front-line skin care products.

Four studies helped answer our next research question, what is the benefit of using tallow on skin? Wozniak et al. looked at the topical application of tallow emulsions with higher content of linoleic acid and reported there was a hydrating property. The higher content of polyunsaturated fatty acids, linoleic acid (C18:2n-6) and alpha-linolenic acid (C18:3n-3), was achieved through enzymatically modifying mutton tallow with hemp seed oil [32]. Mutton tallow naturally contains high amounts of conjugated linoleic acid, which contributes to human health but negligible polyunsaturated fatty acids [38]. Therefore, it was emulsified with hemp seed oil, which primarily contains linoleic acid and alpha-linoleic acid (omega-6 and omega-3, respectively). This emulsion showed a stable and hydrating moisturizer [32]. Overall, this study concluded that the additions of conjugated linoleic acid and polyunsaturated fats to tallow provided emulsions with stability and hydrating properties, which could be of interest to the skincare industry [32]. The enzymatic modification with hemp seed oil created a model system to be expanded upon in the respective field of choice. Another similar study was conducted, which focused on seeing if adding xanthan gum and orange fibers improved the moisturizing properties of the emulsion. However, the study concluded that tallow alone had the most moisturizing impact when applied to the skin [23]. When comparing these results to what we already know about the fatty acid composition of the stratum corneum, palmitic acid C16:0, steric C18:0, palmitoleic acid (C16:1), C18:1, linoleic acid (C18:2), and (all-cis)-11,14,17-eicosatrienoic acid (ETA, C20:3n-3), products that contain higher amounts of linoleic acid should provide benefits. In fact, few articles that we were able to find on the direct uses of tallow emulsions on the skin showed promise of its use and benefits for the skin. Studies such as these, used in combination with the previously reported results on tallow emulsion, can be used to close research gaps through research designs focused directly on the use of tallow on the human skin. Additionally, these results are valuable to understand the use of tallow on human skin, but were both conducted by the same research group. Expanding these methods to different research labs could help expand the knowledge and reproducibility of the results among a larger sample size.

Another study that included moisturizing tests showed that Engkabang fat esters, which are derived primarily from tallow, increased skin hydration. There was an increase of 4.7%, 23.2%, 38.4%, 44.4%, and 47.2% of skin hydration following 30, 60, 90, 120, and 180 minutes of application. However, when Engkabang fat was applied, non-esterfied, there was a decrease of 47.1% in hydration at 30 minutes which may be due to oil being absorbed into the skin and leaving an oily residue on the surface of the skin. However, after this surface layer was absorbed, there was an increase of 8.7% in hydration after 180 minutes. Overall, the tallow fat esters showed significantly more increase in hydration than tallow fat. Furthermore, tallow fat left an oily surface, which may deter certain skin types such as oily or acne-prone skin from using a product with this ingredient [30]. This study supports the need for more research on using tallow in different ways and in different compositions to provide the most beneficial results for users.

The next objective of this paper was to gather research on the therapeutic properties of topically applied tallow. The research results thus far support that tallow is biocompatible with and beneficial to healthy skin, but we also must consider individuals who may suffer from skin diseases or skin that is missing essential properties. Properly hydrating the skin poses a hurdle for many individuals. This issue is further exacerbated by conditions such as dermatitis or psoriasis. A comparative study looked at the effects of a topical emulsion, which had the main ingredient as tallow. Specifically, they concluded that the use of high fat containing emulsions could prove to be helpful in atopic dermatitis or psoriasis. The study included 78 participants, who reported that the moisturizing properties of the emulsion with tallow helped their skin [20]. Another study showed the usefulness of this as it showed that when beef tallow oil was incorporated into a mixture, mice suffering from atopic dermatitis had symptomatic relief. This study also showed the impact of the tallow mixture, which decreased IgE levels in mice that initially had high IgE levels due to pruritus. There was also a decrease in mast cells and B-cell markers [24]. Another therapeutic benefit of topical tallow was wound healing. A study that looked at the benefits of using herbal soap, with tallow as an ingredient, as an anti-infection agent for wound healing versus regular soap. The herbal soap had good efficacy to fight skin infecting bacteria. It was also less drying and damaging to the skin, whereas most chemical soaps tend to dry out the skin. Thus, the herbal soap showed positive results with anti-aging by retaining moisture rather than depriving it [19]. These results indicate that there was an anti-inflammatory impact of a tallow emulsion when applied topically to mice, as well as moisturizing properties during wound healing. This further suggests another research gap in the use of tallow on human skin and how it could show usefulness in inflammatory skin conditions and wound healing.

Additionally, along with the benefits of tallow on skin, we must consider the research gaps on the possible adverse effects of tallow and tallow-derived products. This bring us to the fourth research question: what side effects does tallow have on the skin? As stated previously, tallow is a versatile ingredient in many industries, including the agricultural industry. The process of deriving the fatty acids from animal fats and converting them into amines forms a more amphiphilic product. This composition of both hydrophobicity and hydrophilicity gives the product great versatility to be an ingredient for agricultural products such as herbicides. While this is a very useful tool in the agricultural business, the need to learn about the possible side effects to humans, especially skin, is crucial. POE-T is a surfactant derived from tallow and used in glyphosate herbicides. The use of this non-ionic surfactant is to help with the delivery of glyphosate into plants [39]. Lindberg et al., [25] and Martens et al. [26] explored the effects of tallow amines on skin as it is a common ingredient in agrochemical products. POE-T was not reported to cause significant irritation to a specific organ or body system after repeated exposure through oral and respiratory consumption. However, a GARD skin assay also classified tallow as being a skin sensitizer, which increases the risk of photosensitivity-related irritation [25]. Marten et al. concluded that concentrated POE-T was very irritating to skin, corrosive to the eyes, and sensitizing to the skin. Roughly, a 70% concentrated tallow causes oral, skin, eye, and reproduction toxicity in rats, rabbits, guinea pigs, and dogs. The irritation and sensitization potential of POE-T diminish significantly upon dilution with water. This study also showed that there was severe irritation with 78% of POE-T on the skin of rabbits within 24 hours of contact. However, erythema associated with this resolved within 72 hours. Tallow has been categorized as dangerous and as a compound that can cause serious eye damage as studied in rabbits. However, there is no reproduction toxicity or embryo-fetal development toxicity associated with topical tallow according to this study [26]. Another study showed that tallow in a saline solution when applied topically to intact or abraded skin of rabbits for 24 hours did not cause irritation. Furthermore, when it was applied to their eyes following a rinse or no rinse, there was still no irritation. Also, when applied topically to the skin of guinea pigs, it did not cause any type of hypersensitivity reaction in guinea pigs. This study showed that there was no genotoxicity or carcinogenicity associated with tallow [15]. It was interesting that this study did not perform any testing with tallow on humans, whereas other ingredients in this study were studied on humans. Additionally, Ansell and Fowler classified tallow as being a severe irritant to the skin as it caused erythema and edema. However, it was a moderate irritant to the eye and resulted in corneal effects, and though it did cause iritis and conjunctivitis when applied directly to the eye, it was completely cleared by day 7 [14]. While this study reported tallow as being an irritant to skin and eyes, one must consider the solvent, 2-pyrrolidone, that was used. This is a commonly used solvent in the pharmaceutical and cosmetic industries; thus, we must consider that the irritation may be from the medium used to penetrate the skin and not from tallow itself. Another side effect, which although did not have supporting research but rather was a preventive measure taken by a vaccine manufacturing company, is that tallow when applied topically or given as an infusion may cause prions. The study discussed that vaccines were given to patients with melanoma, which had oleic acid extracted from tallow derived from animal fat. However, the oleic acid was substituted to be extracted from olives due to the fear that there may be a possibility of transmitting prions into the blood from tallow derived from animal fat and causing mad cow’s disease. However, after switching the vaccine, clinical trials showed that the initial vaccine was more effective in melanoma patients. The researchers found that there were less severe reactions with tallow from beef than from olives. The researchers also found there were less severe reactions with the formulation using oleic acid extracted from tallow than from olives. They reported that the possible decrease in reactivity could be due to contamination in the beef-derived formulations, but it was not entirely clear [31]. This article raises two questions - does tallow when applied topically or by other methods cause prion disease, and does tallow have more healing properties when applied topically as compared to directly into the blood stream? Though this was given as an injection directly into the bloodstream, there is concern with topically applied tallow as it still gets absorbed into the blood. Moreover, more research is needed to understand if tallow or other animal-fat derived products can cause something like prion disease.

The evidence about the side effects of tallow and tallow-derived products shows another source of research gaps. The contradicting results supports the need for further research designs that can help establish a standardized safety report on the use of tallow-derived products, such as agricultural products and how these could affect the skin. More research needs to be conducted with controlling variables to determine if it does cause irritation specifically in humans. Though testing on animals may provide insight into how a product performs on human skin, application tests on humans would give more information about which skin types would benefit from the use of tallow and which would not.

Further research into the possible harmful effects of tallow products could also help address the last research question more comprehensively. With the growth of tallow products in the skincare world, the possible effects it could have on the environments that humans commonly encounter is important to consider. Addressing the last objective with the research found, it seems that tallow-derived products are not safe for aquatic life. Personal care products (PCPs) often contain chemicals that may not be harmful topically but can be harmful if ingested. PCPs can often cause harm to the microbiota and are often not safe for aquatic environments. A few papers discussed the effects that tallow has on different aquatic species. The use of POE-T extends to herbicides used in aquatic environments. A study conducted by Haller and Stocker further investigated the effects of ethoxylated tallow amines in herbicides on the scales or skin of bluegill sunfish [18]. When tallow amines were studied in this setting, they were shown to be the most harmful for the fish, suggesting that while deemed safe for human use, they could have negative environmental consequences. Furthermore, another study showed that tallow in aquatic environments has teratogenic effects on microbiota such as Xenopus levis. This may indicate that using tallow topically for an extended period may lead to toxicity in humans, especially to those that have sensitive skin. These results are interesting compared to the research conducted by Martens et al. [26]. These different results on the effects of tallow-derived products may be strictly due to the innate differences in composition of the protective barriers on each study subject. Furthermore, these papers indicate that using tallow amines is not safe for aquatic life; thus, using tallow-based products would raise a concern for people who are looking for environment-friendly options [16].

Overall, there are published studies on the use of tallow that support its use on human skin. However, the reported adverse effects of other tallow-derived products raise concerns about the safety of this product as a main ingredient in certain settings. The research we were able to gather in this scoping review helps add to the small body of research; however, there are significant research gaps. Specifically, there are gaps in the evidence-based knowledge of using tallow directly on the skin. There is a need to continue research on tallow and tallow-derived products and their potential benefits and adverse effects.

Limitations

Some limitations of this study included using only two databases, which produced scarce evidence for tallow providing benefits directly to the skin. This limitation may be due to the difficulty in measuring benefits in non-diseased skin. Upon reviewing the studies available, it seems that when studying topical products, measuring and reporting the adverse effects is a time-saving and efficient route compared to studying the beneficial effects of products over time in a population. However, it can be difficult to assess subjective skin benefits, such as hydration, or to notice an increased absence of adverse events, such as less rashes and irritation. Further databases may have provided more evidence, but this was not explored. Another source of limitations came from the key terms used. The selected terms could have limited the yield of research conducted specifically looking at the effects of tallow on the skin; however, it is possible that there is not a substantial amount of research studying the direct effects of tallow on human skin. More observational studies, case series, prospective studies, and randomized controlled trials are needed to explore many different avenues when it comes to tallow, including studying the effects on both healthy and diseased skin such as patients with atopic dermatitis and psoriasis. Furthermore, limitations came from the sample sizes of studies, the year of publication, and lack of data in humans.

## Conclusions

The purpose of this scoping review was to gather current data on the topical application of tallow. The first objective was to determine how the composition and structure of tallow make it an efficacious agent for the skin. The conclusion was that tallow is primarily composed of oleic acid, palmitic acid, and stearic acid and that tallow increases plasma fatty acid concentration. Topically, it is of notable significance that tallow may increase fatty acid concentration in the skin. The second objective was to analyze the benefits of tallow on the skin. Tallow was found to offer hydrating and moisturizing properties. However, other compounds that were studied in comparative studies such as pumpkin seed oil and linoleic acid were found to offer superior hydrating and moisturizing benefits than tallow. The third objective was to determine the therapeutic properties of topical tallow. Research indicated that tallow may be beneficial in helping with skin conditions such as dermatitis, psoriasis, dry skin, and wounds. However, more research needs to be conducted with larger sample sizes to determine the longitudinal effects of tallow on a variety of skin types and skin disorders. The fourth objective was to analyze the side effects of tallow. This objective resulted in the largest number of discrepancies between papers. Some papers argued that tallow resulted in no irritation to the skin and eyes, others reported that it causes severe irritation, and some only classified either the skin or eyes as having a reaction. The results of these papers showed a significant limitation in understanding any potential adverse side effects of tallow. More research needs to be conducted with a variety of skin types detailing the types of reactions humans have to tallow since all these papers used different measures of reference to document their findings. The fifth and final objective was to determine if tallow is reef-safe. The conclusion we made based on two papers is that it is not reef-safe and is detrimental to marine life. This is important because some consumers prefer products that are environmentally friendly. Tallow is also an animal-derived product, and due to a rising trend through social media and increased awareness about how PCPs are made, many consumers now prefer products that are plant-based or considered vegan, which decreases the marketability of tallow as a skincare or cosmetic ingredient. Moreover, this could also indicate that tallow may have long-term effects on humans as well, which is something that needs more research.

Overall, majority of the objectives in this paper were supported by conclusions made in other papers. After thorough analysis, the side effects of tallow applied topically need to be researched as this is the main gap found with completion of the scoping review. Addressing these side effects and conducting future research that addresses the other objectives will provide more evidence on the efficacy of topical tallow as a skincare ingredient.
